# Comparison of the clinical outcomes of non-invasive ventilation by helmet vs facemask in patients with acute respiratory distress syndrome

**DOI:** 10.1097/MD.0000000000024443

**Published:** 2021-01-29

**Authors:** Mohamad Y. Khatib, Mohamed Z. Peediyakkal, Moustafa S. Elshafei, Hani S. Elzeer, Dore C. Ananthegowda, Muhsen A. Shahen, Wael I. Abdaljawad, Karimulla S. Shaik, Nevin Kannappilly, Ahmed S. Mohamed, Ahmed A. Soliman, Abdulqadir J. Nashwan

**Affiliations:** Critical Care Medicine Department, Hazm Mebaireek General Hospital (HMGH), Hamad Medical Corporation (HMC), Doha, Qatar.

**Keywords:** acute respiratory distress syndrome, coronavirus disease, facemask, helmet, non-invasive ventilation

## Abstract

**Abstract:**

The main aim of this study is to compare the use of non-invasive ventilation (NIV) via helmet versus face mask where different interfaces and masks can apply NIV. However, some of the limitations of the NIV face mask were air leak, face mask intolerance, and requirement of high positive end expiratory pressure, which could be resolved with the use of the helmet NIV. NIV facemask will be applied as per the facial contour of the patient. NIV helmet is a transparent hood and size will be measured as per the head size. Both groups will have a standard protocol for titration of NIV.

Patients aged more than 18 years old and diagnosed with acute respiratory distress syndrome as per Berlin definition will be enrolled in the study after signing the informed consent. Subjects who met the inclusion criteria will receive 1 of the 2 interventions; blood gases, oxygenation status [Po2/Fio2] will be monitored in both groups. The time of intubation will be the main comparison factor among the 2 groups. The primary and secondary outcomes will be measured by the number of patients requiring endotracheal intubation after application of helmet device, Improvement of oxygenation defined as PaO2/FiO2 ≥ 200 or increase from baseline by 100, duration of mechanical ventilation via an endotracheal tube, intensive care unit length of stay, death from any cause during hospitalization at the time of enrolment, need for proning during the hospital stay, intensive care unit mortality, and the degree to which overt adverse effects of a drug can be tolerated by a patient including feeding tolerance.

**Trial registration number:**

NCT04507802.

**Protocol version:**

May 2020

## 1 Introduction

Pneumonia is one of the leading causes of intensive care unit (ICU) admissions. Among pneumonia patients, acute respiratory distress syndrome (ARDS) is the most common etiological factor leading to respiratory failure. Since March 2020, worldwide ICU admissions were mainly ARDS patients infected with coronavirus disease (COVID-19).

Within intensive care units, approximately 10% to 15% of admitted patients, and up to 20% of mechanically ventilated patients meet ARDS criteria.^[1–3]^

In Qatar, between February 28 and April 18, 2020, 5685 cases of COVID-19 were identified. More than 90% were with minimal symptoms or asymptomatic, with 2.0% required critical care support.^[4]^ COVID-19 patients with existing comorbidities (mainly hypertensive and diabetes) had higher C-reactive protein (CRP) levels (inflammatory response), a higher ICU admission rate, extended hospitalization, and more oxygen use.^[5,6]^ On the other hand, patients with eosinophilia had a lower level of CRP, milder clinical course and better disease outcomes compared to those without eosinophilia.^[7]^

The Berlin definition of ARDS requires that all the following criteria be present for diagnosis.^[8,9]^

1. Respiratory symptoms must have begun within 1 week of a known clinical insult, or the patient must have new or worsening symptoms during the past week.
2. Bilateral opacities must be present on a chest radiograph or computed tomographic (CT) scan. These opacities must not be fully explained by pleural effusions, lobar collapse, lung collapse, or pulmonary nodules.
3. The patient's respiratory failure must not be fully explained by cardiac failure or fluid overload. An objective assessment (echocardiography) to exclude hydrostatic pulmonary edema is required if no risk factors for ARDS are present.
4. A moderate to severe impairment of oxygenation must be present, as defined by the ratio of arterial oxygen tension to the fraction of inspired oxygen (PaO_2_/FiO_2_). The severity of the hypoxemia defines the severity of the ARDS:
  - Mild ARDS – The PaO_2_/FiO_2_ is >200 mm Hg, but ≤300 mm Hg, on ventilator settings that include positive end-expiratory pressure (PEEP) or continuous positive airway pressure (CPAP) ≥5 cm H_2_O.
  - Moderate ARDS – The PaO_2_/FiO_2_ is >100 mm Hg, but ≤200 mm Hg, on ventilator settings that include PEEP ≥5 cm H_2_O.
  - Severe ARDS – The PaO_2_/FiO_2_ is ≤100 mm Hg on ventilator settings that include PEEP ≥5 cm H_2_O

Acute respiratory distress syndrome (ARDS) is characterized by a progressive form of respiratory failure with hypoxia, bilateral lung infiltrates on chest imaging, and onset within a week of the triggering event. There are many common etiological factors for ARDS which can be classified into direct and indirect lung injury factors. The commonest direct lung injury cause is pneumonia which includes both bacterial and viral.

The pathology of ARDS lung evolves through 3 phases with inflammatory changes. The inflammation leads to increased microvascular permeability which leads to extravascular fluid accumulation. There will be interstitial and alveolar edema which progresses to fibrosis. The characteristic lesion termed diffuse alveolar damage, undergoes progression from an exudative to proliferative, to a fibrotic phase. Histologic studies in ARDS have demonstrated a pattern of diffuse pulmonary endothelial injury associated with both macro and microscopic thrombi formation. These early changes progress to fibro cellular intimal proliferation that can obliterate small vessels.^[10]^

The treatment of ARDS is a field where there are lots of researches being done. Until now, the treatment modalities that have shown mortality benefit in ARDS are neuromuscular blockade, prone ventilation and extracorporeal membrane oxygenation. There are few recent studies in ARDS treatment using NIV to avoid endotracheal intubation and mechanical ventilation-induced adverse events. The main side effects associated with mechanical ventilation include the need for sedation and paralysis, barotrauma, critical illness polyneuropathy. The non-invasive mechanical ventilation (NIV) is any form of administration of positive pressure using an interface or a face mask, without the need of using an endotracheal tube.^[11]^

Hypoxemic acute respiratory failure is the most common form of acute respiratory failure associated with ARDS. The application of positive pressure in hypoxemic acute respiratory failure produces decreased intrapulmonary shunt and thereby improves oxygenation. This occurs by the recruitment of collapsed alveoli, prevents collapse previously opened by the positive pressure, increases FRC, and improves the balance of the ventilation-perfusion mismatch.^[12,13]^

There are also some studies which showed benefit with the use of NIV in community-acquired pneumonia patients.^[8,14]^

NIV can be applied by different interfaces and masks. The limitations of the face mask NIV that are found in some studies were air leak, face mask intolerance, and requirement of high PEEP.^[15–17]^

These obstacles were overcome to some extent by the introduction of helmet NIV. It provided better patient tolerance, less air leak as there is no contact with the facial tissues, and a better seal at the neck level. To our knowledge, there is only 1 randomized trial that compared the effects of helmet NIV versus face mask NIV in preventing endotracheal intubation.^[18]^ The results of the trial showed the intubation rate was 61.5% (n = 24) for the face mask group and 18.2% (n = 8) for the helmet group (absolute difference, –43.3%; 95% CI, –62.4% to –24.3%; *P* < .001). As evidenced from the mentioned trial, we hypothesize the use of an NIV helmet can reduce intubation in ARDS caused by pneumonia, including COVID-19.

## 2 Objectives

Primary objective: To assess the efficacy of non-invasive ventilation with HELMET in reducing endotracheal intubation rates in comparison with NIV face mask among patients with ARDS.

Secondary objective: to explore any improvement in oxygenation, time to intubation, ventilation days, and tolerance to HELMET NIV in comparison with facemask NIV.

## 3 Trial design

This is a phase-III, randomized, comparative, parallel assignment, open-label clinical study

## 4 Methods: participants, interventions, and outcomes

### 4.1 Study setting

The study will take place in the intensive care unit in Hazm Mebaireek General Hospital in Qatar. All included patients are diagnosed with ARDS, type 1, or type 2 respiratory failure, and they are conscious and alert. All the investigators on this protocol are from Hazm Mebaireek General Hospital. The details of the procedure and interventions are mentioned below in the background and methods sections.

### 4.2 Eligibility criteria

1. Patients with ARDS as per Berlin definition
2. Age: 18 years and above

### 4.3 Exclusion criteria

1. Patients with altered sensorium [Glasgow Coma Scale less than 13]
2. Pregnant patients
3. Hemodynamic instability
4. Morbidly obese
5. Patients with tracheostomy
6. Severe acidosis [PH less than 7.15]
7. Patients with glaucoma
8. Patients with a history of vertigo

### 4.4 Outcomes measures

Primary outcome measure:

1. Need for endotracheal intubation [ time frame: 6 weeks].
  - Number of patients requiring endotracheal intubation after application of helmet device

Secondary outcome measures:

1. Improvement in oxygen saturation [time frame: 2 weeks]
  - Improvement of oxygenation defined as “PaO2/FiO2 ≥ 200 or increase from baseline by 100”.
2. Ventilator-free days [time frame: 28 days]
  - Duration of mechanical ventilation via endotracheal tube.
3. Intensive care unit length of stay [time frame: 4 weeks]
  - The number of days admitted to the intensive care unit.
4. Overall mortality [time frame: 90 days]
  - Death from any cause during hospitalization at the time of enrollment.
5. Need for proning [time frame: up to 24 weeks]
  - Need for proning during the hospital stay.
6. ICU mortality [time frame: 28 days]
  - Death from any cause during ICU hospitalization at the time of enrollment.
7. Patient tolerability [time frame: 28 days from randomization]
  - The degree to which overt adverse effects of a drug can be tolerated by a patient, including feeding tolerance.

### 4.5 Participant timeline

The expected time of the trial will be 1 calendar year after ethical approval and will be renewed annually for 5 years.

### 4.6 Sample Size & Recruitment

Sixty patients will be recruited after randomizing the selected patients with odd or even numbers.

## 5 Methods: data collection, management, and analysis

### 5.1 Type and classification of study- randomized control trial

*Intervention* – application of NIV by HELMET after randomizing the selected patients with odd and even numbers.

*Control group-* application of NIV with face mask after randomizing the selected patients with ARDS caused by COVID-19 and other pneumonia, type 1 and type 2 respiratory failure, Conscious and alert patients. NIV facemask will be applied as per the facial contour of the patient. NIV helmet is a transparent hood and size will be measured as per the head size. Both groups will have a standard protocol for titration if NIV. First, PEEP will be increased gradually to target spo2 above 90% with a Fio2 of 60% or less. Pressure support or IPAP will be increased slowly by 2 to 3 cm H2O to target a respiratory rate less than 30/min. The patient will be connected to non-invasive ventilation for 4 hours and will be on a high-flow nasal cannula for 1 hour. This 4:1 ratio will be continued till his condition improves or he will get intubated if he is worsening on non-invasive ventilation. Blood gases, oxygenation status [Po2/Fio2] will be monitored in both groups. The time of intubation will be the main comparison factor among the 2 groups. The intubation criteria will be the same for 2 groups which include, Worsening P/F ratio worsening acidosis, hemodynamic instability, poor neurological status.

The decision for intubation will be decided by the primary physician on duty

The intubated patient will be ventilated with lung-protective ventilation as per ARDS protocol.

If a patient in the control group is not tolerating the mask, they will be given the helmet in the other group as rescue therapy.

The weaning criteria will be the same for both groups and patients will be weaned to a high-flow nasal cannula or non-rebreathing mask. For weaning, pressure support and PEEP will be reduced gradually and will be discontinued if the respiratory rate is less than 30/min and P/F ratio more than 150 with a PEEP of 5 cm H20. If 1 patient is out of NIV for more than 12 hours a day for 48 hours, that will be considered as weaning.

### 5.2 Data management

The responsibilities of the designated study team include project compliance, data collection, abstraction and entry, data reporting, regulatory monitoring, problem resolution and prioritization, and coordination of the activities of the protocol study team.

The collected data for this study will be transferred to a secure database managed by the HMC IT team (e.g., PACS). All data generated in this study will be the property of HMC.

Source documentation will be available to support the computerized patient record. Study personnel will record clinical data in each patient's source documents (i.e., the patient's medical record). The study team will maintain adequate and accurate records to enable the conduct of the study to be fully documented and the study data to be subsequently verified. After study closure, the investigators will maintain all source documents, study-related documents, and the data stored in the database used for data collection. Data will be entered throughout the trial as patients are enrolled.

### 5.3 Statistical methods

Data analysis will be carried out by the principal investigator with the help of a biostatistician. Descriptive statistics will be used to summarize and determine the sample characteristics and distribution of parameters related to demographic, presenting signs and symptoms, laboratory findings. The normally distributed data and results will be reported with mean and standard deviation with corresponding 95% confidence interval.

## 6 Methods: monitoring

### 6.1 Data monitoring & auditing

The clinical trial master file will be kept along with informed consent from the patients according to the Joint Commission International, Medical Research Center (MRC), and the Ministry of Public Health (MoPH) requirements.

Any side effects or adverse events related to the trial will be reported to the hospital research committee and MRC.

A regular registration report will be generated to monitor patient accrual and completeness of registration data. A routine data quality report will be generated to assess missing data and inconsistencies. Accrual rate and extent and accuracy of evaluations and follow-up will be monitored periodically throughout the study period, and potential problems will be brought to the attention of the study team for discussion and action. Random-template data quality and protocol compliance audits may be conducted by the study team, at a minimum of once per year or more frequently if indicated. Data safety and monitoring will be conducted according to the HMC-IRB, Ethics, and Data Safety Monitoring Board regulations.

## 7 Ethics and dissemination

### 7.1 Research ethics approval

The study will be conducted in full conformance with principles of the “Declaration of Helsinki”, Good clinical practice (GCP) and within the laws and regulations of MoPH in Qatar.

### 7.2 Protocol amendments

None

### 7.3 Informed consent

The researcher will do screening from the patient pool; those patients found to be eligible as per inclusion and exclusion criteria will be interviewed to discuss the trial if accepted, they will be enrolled in the study the PI where the participant will be given free time to decide to participate or not the consenting process will start by screening then interviewing the eligible patient explaining about the trial rationale benefits risks and objectives his or her right to participate or not to participate without being affected and the right to withdraw at any time.

### 7.4 Confidentiality

Patients’ data will be coded and kept in a secure database with a unique username and password to maintain patient confidentiality. Only the authorized research team will be granted access to the patients’ electronic charts and reports.

### 7.5 Declaration of interests

None

### 7.6 Access to data

Data collected in this study will be transferred to a secure database managed by the HMC IT team (e.g., PACS). All data generated in this study will be the property of HMC.

### 7.7 Dissemination policy

The findings of this trial will be published as jointly co-authored manuscripts in international medical journals subject to peer review and deposited into the US National Library of Medicine made freely available to the public.

## Author contributions

**Conceptualization:** Mohamad Y. Khatib, Mohamed Z. Peediyakkal.

**Methodology:** Mohamad Y. Khatib, Mohamed Z. Peediyakkal, Moustafa S. Elshafei, Hani S. Elzeer, Dore C. Ananthegowda, Muhsen A. Shahen, Wael I. Abdaljawad, Karimulla S. Shaik, Nevin Kannappilly, Ahmed S. Mohamed, Ahmed A. Soliman, Abdulqadir J. Nashwan.

**Project administration:** Abdulqadir J. Nashwan.

**Writing – original draft:** Mohamad Y. Khatib, Mohamed Z. Peediyakkal, Moustafa S. Elshafei, Hani S. Elzeer, Dore C. Ananthegowda, Muhsen A. Shahen, Wael I. Abdaljawad, Karimulla S. Shaik, Nevin Kannappilly, Ahmed S. Mohamed, Ahmed A. Soliman, Abdulqadir J. Nashwan.

**Writing – review & editing:** Mohamad Y. Khatib, Mohamed Z. Peediyakkal, Moustafa S. Elshafei, Hani S. Elzeer, Dore C. Ananthegowda, Muhsen A. Shahen, Wael I. Abdaljawad, Karimulla S. Shaik, Nevin Kannappilly, Ahmed S. Mohamed, Ahmed A. Soliman, Abdulqadir J. Nashwan.

## Abbreviations

Abbreviations: ARDS = acute respiratory distress syndrome, COVID- 19 = coronavirus disease, ICU = intensive care unit, NIV = non-invasive ventilation, PEEP = positive end expiratory pressure.
